# The Diabetes Health Plan and Healthcare Utilization Among Beneficiaries with Low Incomes

**DOI:** 10.1007/s11606-022-07903-9

**Published:** 2022-11-16

**Authors:** Kimberly Danae Cauley Narain, Norman Turk, O. Kenrik Duru, Tannaz Moin, Carol M. Mangione

**Affiliations:** 1 Division of General Internal Medicine and Health Services Research (GIM/HSR), Department of Medicine, David Geffen School of Medicine, University of California, 1100 Glendon Ave., Suite 850, Los Angeles, CA 90024 USA; 2 Center for Health Advancement, Fielding School of Public Health, University of California Los Angeles, Los Angeles, CA USA; 3 VA Greater Los Angeles Healthcare System, Los Angeles, CA USA; 4 Fielding School of Public Health, University of California, Los Angeles, CA USA

**Keywords:** diabetes, low income, healthcare utilization, value-based insurance design

## Abstract

**Background:**

The socioeconomic status (SES) gradient in hospital and emergency room utilization among adults with type 2 diabetes (T2DM) is partially driven by cost-related non-adherence.

**Objective:**

To test the impact of the Diabetes Health Plan (DHP), a diabetes-specific health plan incorporating value-based insurance design principles on healthcare utilization among low-income adults with T2DM.

**Design:**

To examine the impact of the DHP on healthcare utilization, we employed a difference-in-differences (DID) study design with a propensity-matched comparison group. We modeled count and dichotomous outcomes using Poisson and logit models, respectively.

**Participants:**

Cohort of adults (18–64) with T2DM, with an annual household income <$ 30,000, and who were continuously enrolled in an employer-sponsored UnitedHealthcare plan for at least 2 years between 2009 and 2014.

**Interventions:**

The DHP reduces or eliminates out-of-pocket costs for disease management visits, diabetes-related medicines, and diabetes self-monitoring supplies. The DHP also provides access to diabetes-specific telephone case management as well as other online resources.

**Main Measures:**

Number of disease management visits (*N* = 1732), any emergency room utilization (*N* = 1758), and any hospitalization (*N* = 1733), within the year.

**Key Results:**

DID models predicting disease management visits suggested that DHP-exposed beneficiaries had 1.7 fewer in-person disease management visits per year (− 1.70 [95% CI: − 2.19, − 1.20], *p* < 0.001), on average, than comparison beneficiaries. Models for emergency room (0.00 [95% CI: − 0.06, 0.06], *p* = 0.966) and hospital utilization (− 0.03 [95% CI: − 0.08, − 0.01], *p* = 0.164) did not demonstrate statistically significant changes associated with DHP exposure.

**Conclusions:**

While no relationship between DHP exposure and high-cost utilization was observed in the short term, fewer in-person disease management visits were observed. Future studies are needed to determine the clinical implications of these findings.

## INTRODUCTION

The prevalence of diabetes has increased over the past two decades in the USA, disproportionately affecting populations with low incomes. Between 2011 and 2014, compared with persons with high income, the relative percentage increase in diabetes prevalence was 40.0%, 74.1%, and 100.4% for those classified as middle income, near poor, and poor, respectively.^1^ Furthermore, studies have found a socioeconomic gradient in diabetes-related complications and healthcare utilization.^2^ Non-adherence to medications and treatment recommendations due to cost is an important driver of the socioeconomic status (SES) gradient in morbidity among adults with diabetes.^3^ Although isolated copayments for medications and medical visits may be low, taken in the aggregate, these costs may pose a financial burden for individuals with low incomes, forcing tradeoffs between medical care and basic necessities.^4^ Consequently, patients may opt to forgo needed treatments, leading to diabetes-related complications. Cross-sectional studies have demonstrated a negative relationship between medication adherence, emergency room utilization, and hospitalizations.^5–7^

Methodologically rigorous studies suggest that health insurance plans that incorporate value-based insurance design (VBID) principles such as lowering out-of-pocket costs for medications used to treat chronic disease may be particularly effective for improving medication adherence among patients with low SES, but few studies have evaluated the healthcare utilization effects of such plans among this subpopulation. Choudhry et al. found that a randomized control trial in which patients with a recent myocardial infarction were randomized to either a health insurance plan that eliminated co-insurance, copayments for disease management visits, and copayments for secondary prevention medications, or usual health insurance coverage, reduced racial/ethnic disparities in major vascular events or revascularization among the intervention group.^8^ Observational studies of the utilization implications of health insurance plans incorporating VBID principles among the broader patient population have demonstrated reductions in emergency room utilization and hospitalization among these beneficiaries; however, it is not clear that these findings can be extrapolated to populations with low SES.^9^ There may be differences in health literacy, self-management knowledge, access to primary care, and other social determinants of health that translate into different healthcare utilization implications of these types of health insurance plans among the subpopulation with low socioeconomic status.^10^ Given the upfront costs to insurers of incorporating VBID principles into health insurance plans and associated costs due to increases in medication adherence, it will be important to have information regarding how healthcare utilization may be impacted among one of the most affected subpopulations of beneficiaries. The objective of this study is to examine the impact of the Diabetes Health Plan (DHP), (the first condition-specific health insurance plan based on VBID principles), on healthcare utilization among beneficiaries with low household incomes (< $30 K annually).

In 2009, UnitedHealthCare (UHC) introduced the DHP which includes financial incentives to encourage patient engagement in evidence-based diabetes care, including reduced or eliminated out-of-pocket patient expenses for disease management visits; free diabetes self-monitoring training and supplies; and reduced or eliminated out-of-pocket expenses for diabetes-related medicines (Table 1).^11,12^ The DHP also provides access to diabetes-specific telephone case management as well as other online resources. Additionally, the DHP provides scorecards with reminders to complete health maintenance activities, such as biannual hemoglobin A1C and cholesterol screening and an annual retinal eye exam. Overall, the DHP provides between $150 and 500 in annual out-of-pocket savings for enrollees.^13^ The DHP standard benefit design can be modified by purchasing employers to better suit the needs of beneficiaries, which include both employees and their dependents. Uptake of the DHP, across employers, has spanned the years since 2009 and varied across years. Some employers use an opt-in enrollment strategy (employees must choose to participate) while others use an opt-out strategy (all eligible employees enrolled initially). Studies have shown that DHP uptake can range from a low of 8% among opt-in plans to a high of 85% for opt-out plans.^14^ Additionally, studies have shown variability in the demographic characteristics of DHP participants as a consequence of the enrollment strategy. Specifically, Kimbro et al. found that DHP participants enrolled in an opt-out plan were more likely to be dependents, were more racially and ethnically diverse, and had a broader range of incomes and educational backgrounds relative to participants enrolled in opt-in plans, who tended to have higher incomes and more education and who were less likely to be Hispanic.^12^ Studies have previously demonstrated the beneficial impact of the DHP on medication adherence and emergency room utilization among the broader cohort of beneficiaries.^13,15^ However, the results of studies examining the effects of the DHP among low-income beneficiaries have been mixed. Huang et al. found no relationship between the DHP and medication adherence among beneficiaries with household incomes of $50,000 or less.^16^ While Narain et al. found that the DHP was associated with improved adherence to oral hypoglycemic medications among beneficiaries with household incomes $30,000 or less and low baseline medication adherence.^17^

**Table 1:** Features and Costs by DHP and Standard Plan

Feature	DHP	Standard plan
Office visit copays		
Primary care	$0	$20
Specialist visits	$0–10	$30
Premium cost to the employee	Standard	Standard
Prescription copays		
Metformin, statins, ACE/ARB, etc.	$0	$5–15
Lab tests	Covered	Covered
Online tracking	Included	Availability varied
Diabetes disease management	Included	Availability varied
Weight management	Included	Availability varied

## METHODS

### Data Source and Population

The analytic data set is limited to 26 large employer groups that purchased the DHP and standard benefit plans from UHC (2009–2014) that have (1) internal pharmacy contracts, (2) complete pharmacy claims data, (3) sufficient medical claims and lab data to identify employees with type 2 diabetes (T2DM), and (4) fewer than 15% of employees enrolled in high deductible health plans. In addition to the above-mentioned criteria, the DHP employer groups must have at least 1 year of standard benefit plan data, prior to the purchase of the DHP, and comparison employer groups are further limited to those that have overlapping propensity scores with DHP employers after employer-level matching (described further below) and who have at least 2 years of continuous enrollment in the standard benefit plan during the duration of the match to the DHP employer.

**Figure 1:**
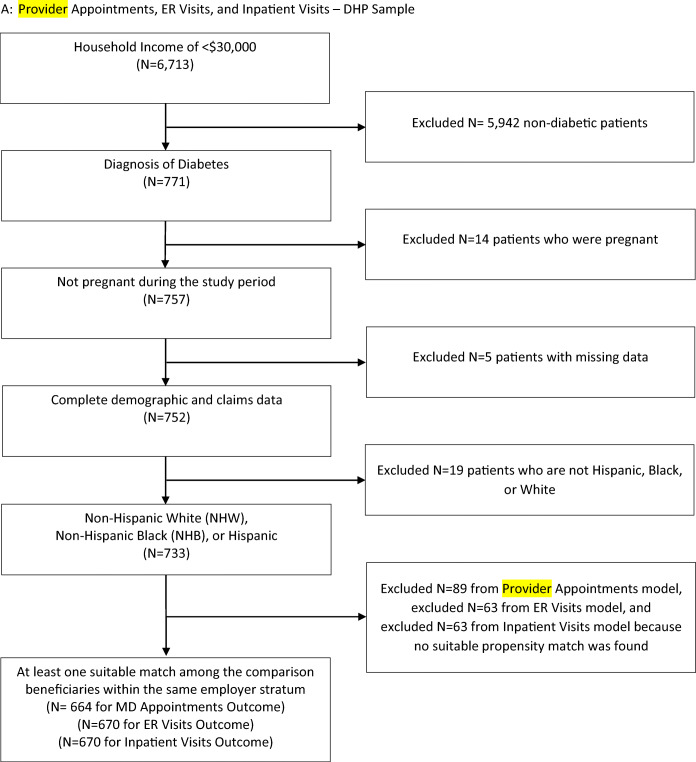
Provider appointments, ER visits, and inpatient visits—DHP sample.

**Figure 2:**
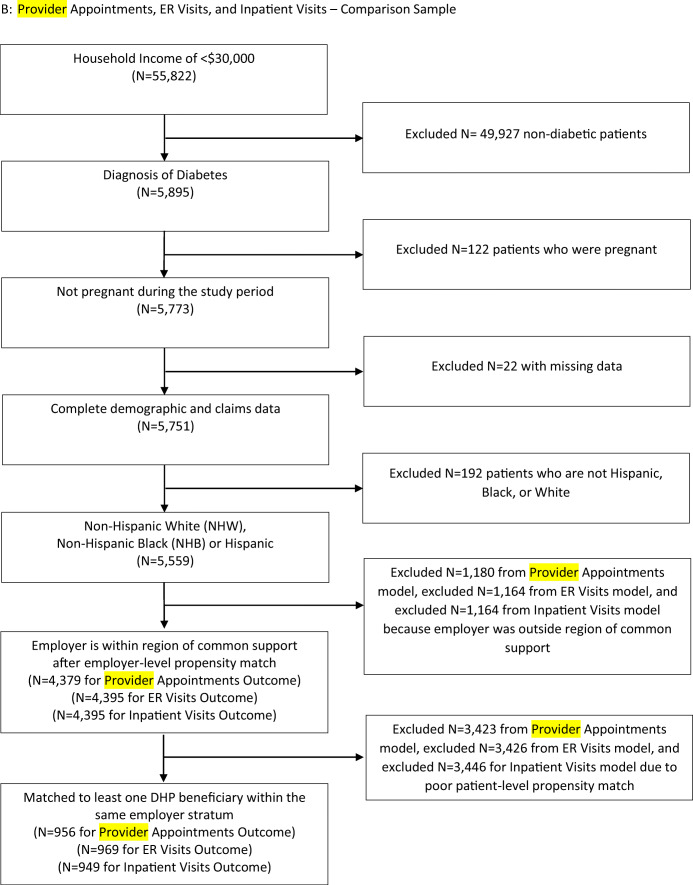
Provider appointments, ER visits, and inpatient visits—comparison sample.

A diabetes diagnosis was defined as having any of the following prior to the implementation of the DHP: (1) at least one 250.X ICD-9 diagnosis code from an inpatient, outpatient, or emergency department claim; (2) hemoglobin A1C laboratory value of 6.5% or greater or a 2-h value on an oral glucose tolerance test of greater than 200 mg/dl; or (3) at least one prescription fill for an oral hypoglycemic medication other than metformin or insulin. Estimated household income is obtained from the *AmeriLINK data.*^18^ This data source incorporates consumer financial survey responses, publicly available information (public records, census information, and retail transaction records), and zip-code-level information from the Internal Revenue Service to generate individual-level estimates of household income. The sample size flow chart for the unique DHP and comparison beneficiaries are shown in Figures 1 and 2, respectively.

### Propensity Score Matching

Matching criteria for both the DHP and comparison employers were derived with respect to the 12-month period preceding the date of DHP adoption for the DHP employers or standard insurance plan contract renewal (the index date). The matching criteria included the following as reported by UHC: average employee salary, geographic region, number of employees, % female, % in each racial/ethnic category (White, Black, Asian, Hispanic), health benefit plan generosity, % of employees with a HDHP, and % of beneficiaries with each one of the following claims-based co-morbidities (hypertension, hyperlipidemia, coronary artery disease, anxiety/depression, dementia, osteoarthritis, rheumatoid arthritis, non-skin cancer, chronic obstructive pulmonary disease, congestive heart failure, atrial fibrillation, end-stage renal disease, peripheral vascular disease, stroke, schizophrenia) as well as the index date. A single comparison employer could be matched to more than one DHP employer. Individual-level matching criteria were based on the following pre-index date criteria: race and ethnicity, age, gender, Charlson Comorbidity Index, insulin use status, presence of any diabetes complication (retinopathy, nephropathy, neuropathy, cardio/peripheral vascular disease, history of a diabetes-related hospitalization), and baseline healthcare utilization. Nearest-neighbor matching was conducted with replacement using a caliper equal to 25% of the propensity score standard deviation, in an effort to get 3 comparison matches for each DHP beneficiary.^19^ The employer and beneficiary matching was done using PROC PSMATCH in SAS version 9.4.

### Outcomes

We coded “disease management visits” as a count variable based on the composite number of outpatient visits with providers who may perform diabetes management during the course of a visit (endocrinologist, internal medicine, family practice, urgent care specialist, nurse practitioner, physician assistant). Patients with an unusually high number of disease management appointments in the baseline year were excluded from this analysis prior to matching using the 1.5 interquartile range heuristic for identifying outliers.^20^ We treated emergency room and hospital utilization as dichotomous variables. Both variables were indicators coded as “1” if the utilization was present during the post-period year and coded as “0” if the utilization was not present. These utilization outcomes were not restricted to those exclusively related to diabetes.

### Statistical Analyses

We used a DID study to examine the impact of the DHP on utilization. The key assumption of the DID study is the parallel trends assumption which necessitates that the pre-intervention trends for outcome measures across the treatment and comparison groups are the same.^21^ If the parallel trend assumption is met, any difference in the pre-post intervention change in slope across treatment and comparison groups is attributed to intervention effects. We use the propensity-matched sample to increase the likelihood that the DHP and comparison groups have a similar trend of utilization during the pre-intervention time period.^22^ Non-linear statistical models were run for each of the utilization outcomes using the PROC GENMOD procedure in SAS. The model used for disease management visits employed a Poisson distribution with a log link function, and binomial logit models were used to model emergency room and hospital utilization. These models include an indicator for time (post-index vs. pre-index) that was coded as “1” if the observation was from the post-index year and coded as “0” if the observation was from the pre-index (baseline) year, and an indicator for group (DHP group vs. comparison group) that was coded as “1” if the observation was from the DHP group and coded as “0” if the observation was from the comparison group and the interaction between time and group, among our matched samples. Specifically, the between-group differences in the change of the outcome variables, post-index, were estimated by the interaction effects.

### Sensitivity Analyses

We conducted an additional test to assess the sensitivity of our results to selection bias by repeating the above-mentioned analyses with DHP employers that use an opt-out enrollment strategy as the sole source of the treatment population. This methodological change should allow for evaluation of the DHP utilization effects among a less motivated subset of beneficiaries than the subset including individuals that proactively enrolled in the DHP.^12^

## RESULTS

The final analytic samples were 1732, 1758, and 1733 matches with replacement for disease management visits, emergency room use, and hospitalizations, respectively. Across all three samples, prior to matching, the DHP sample was older, had a higher proportion of females, was more likely to be non-Hispanic Black, was more likely to have a diabetes complication, and had a higher Charleston Co-morbidity Index (Tables 2, 3 and 4). Post-matching mean standardized differences for all covariates included in the propensity score models are < 0.1, across the DHP and comparison beneficiaries, indicating sufficient matching.^19^

**Table 2:** Comparison of Treatment and Control Samples for the Disease Management Visit Count Model Before and After Propensity Score Matching

Covariates	DHP sample	Comparison sample prior to matching		Comparison sample after propensity score matching		Std. mean differences after matching^*^
	*N* = 664	*N* = 4379	*p* value	*N* = 1068	*p* value	
Mean age (SD)	53.5 (8.9)	51.5 (9.8)	< .001	52.6 (7.5)	.038	.12
Female	59.2%	52.1%	< .001	60.0%	.766	.02
Race/ethnicity						
Hispanic	17.6%	30.1%	< .001	18.2%	.918	< .01
Black	39.8%	24.2%		38.7%		
White	42.6%	45.7%		43.1%		
Insulin use	19.1%	18.0%	.481	19.3%	.926	< .01
Diabetes Complication Index	42.5%	35.6%	< .001	45.0%	.347	.06
Charleston Co-morbidity Index (SD)	1.8 (1.5)	1.7 (1.3)	.009	1.8 (1.1)	.762	.02
Unadjusted appointment count at baseline (SD)	5.8 (4.4)	5.7 (4.5)	.614	5.8 (4.4)	.712	.02

Propensity scores were generated using logistic regression models that included the following pre-index date criteria: race/ethnicity, age, gender Charleston Co-morbidity Index, Diabetes Complication Index (presence of any diabetes complication (retinopathy, nephropathy, neuropathy, cardio/peripheral vascular disease, history of a diabetes-related hospitalization)), insulin usage, and baseline count of provider appointments. Nearest-neighbor matching was conducted with replacement using a caliper of 25% SD of the propensity score in an effort to get 3 comparison matches for each DHP-exposed beneficiary. Bivariate *p* values were generated using *t* test and chi-squared test for continuous and categorical/dichotomous variables, respectively. *N* = number of matches ≠ number of individuals for comparison beneficiaries

^*^Absolute value of standardized mean differences between the DHP and propensity-matched sample

**Table 3:** Comparison of Treatment and Control Samples for the Emergency Room Model Before and After Propensity Score Matching

Covariates	DHP sample	Comparison sample prior to matching		Comparison sample after propensity score matching		Std. mean differences after matching^*^
	*N* = 670	*N* = 4395	*p* value	*N* = 1088	*p* value	
Mean age (SD)	53.5 (8.9)	51.5 (9.8)	< .001	52.8 (7.3)	.109	.09
Female	59.6%	52.2%	< .001	57.8%	.518	.04
Race/ethnicity						
Hispanic	17.6%	30.2%	< .001	18.3%	.944	< .01
Black	40.0%	24.1%		39.8%		
White	42.4%	45.7%		41.8%		
Insulin use	19.4%	18.0%	.364	18.2%	.560	.03
Diabetes Complication Index	43.0%	35.7%	< .001	43.2%	.935	< .01
Charleston Co-morbidity Index (SD)	1.8 (1.5)	1.7 (1.3)	.004	1.8 (1.1)	.964	< .01
Unadjusted appointment count at baseline (SD)	0.51 (1.12)	0.43 (1.0)	.099	0.49 (0.73)	.766	.02

Propensity scores were generated using logistic regression models that included the following pre-index date criteria: race/ethnicity, age, gender Charleston Co-morbidity Index, Diabetes Complication Index (presence of any diabetes complication (retinopathy, nephropathy, neuropathy, cardio/peripheral vascular disease, history of a diabetes-related hospitalization)), insulin usage, and baseline count of ER visits. Nearest-neighbor matching was conducted with replacement using a caliper of 25% SD of the propensity score in an effort to get 3 comparison matches for each DHP employee. Bivariate *p* values were generated using *t* test and chi-squared test for continuous and categorical/dichotomous variables, respectively. *N* = number of matches ≠ number of individuals for comparison beneficiaries

^*^Absolute value of standardized mean differences between the DHP and propensity-matched sample

**Table 4:** Comparison of Treatment and Control Samples for the Inpatient Model Before and After Propensity Score Matching

Covariates	DHP sample	Comparison sample prior to matching		Comparison sample after propensity score matching		Std. mean differences after matching^*^
	*N* = 670	*N* = 4395	*p* value	*N* = 1063	*p* value	
Mean age (SD)	53.5(8.9)	51.5 (9.8)	< .001	53.2 (7.1)	.466	.04
Female	59.6%	52.2%	< .001	58.4%	.657	.02
Race/ethnicity			< .001		.866	.02
Hispanic	17.6%	30.2%		18.3%		
Black	40.0%	24.1%		40.7%		
White	42.4%	45.7%		41.0%		
Insulin use	19.4%	18.0%	.364	17.0%	.257	.06
Diabetes Complication Index	43.0%	35.7%	< .001	43.4%	.871	< .01
Charleston Co-morbidity Index (SD)	1.8 (1.5)	1.7 (1.3)	.004	1.8 (1.2)	.824	.01
Unadjusted appointment count at baseline (SD)	1.37 (6.92)	0.88 (5.20)	.081	1.10 (4.1)	.391	.05

Propensity scores were generated using logistic regression models that included the following pre-index date criteria: race/ethnicity, age, gender Charleston Co-morbidity Index, Diabetes Complication Index (presence of any diabetes complication (retinopathy, nephropathy, neuropathy, cardio/peripheral vascular disease, history of a diabetes-related hospitalization), insulin usage, and baseline count of inpatient visits. Nearest-neighbor matching was conducted with replacement using a caliper of 25% SD of the propensity score in an effort to get 3 comparison matches for each DHP employee. Bivariate *p* values were generated using *t* test and chi-squared test for continuous and categorical/dichotomous variables, respectively. *N* = number of matches ≠ number of individuals for comparison beneficiaries

^*^Absolute value of standardized mean differences between the DHP and propensity-matched sample

DID models predicting disease management visits suggested that DHP-exposed beneficiaries had 1.7 fewer disease management visits per year, on average, than comparison beneficiaries ([95% CI: − 2.19, − 1.20], *p* < 0.001; Table 5). Models for emergency room (0.00 [95% CI: − 0.06, 0.06], *p* = 0.966) and hospital utilization (− 0.03 [95% CI: − 0.08, − 0.01], *p* = 0.164) did not demonstrate statistically significant changes associated with DHP exposure. In sensitivity analyses including only employers enrolling DHP beneficiaries using an opt-out strategy as the treatment population, once again, we find a negative association between DHP exposure and disease management visits, with DHP beneficiaries visiting providers 2.0 fewer times, on average, 1 year after exposure compared to controls (95% CI: − 2.7, − 1.4, *p* < 0.001), but no association with emergency room (− 0.05 [95% CI: − 0.13, 0.02], *p* = 0.165) or hospital utilization (0.00 [95% CI: − 0.06, 0.06], *p* = 0.982; Table 6).

**Table 5:** Predicted Change with DHP Exposure, Relative to No Exposure, for the Opt-In Samples (Difference in Differences)

Utilization measure	Disease management visit count		Percent with any ER visit		Percent with any hospitalization	
Unadjusted vs. adjusted	Unadj. means (SE)	Adjusted differences (CI)	Unadj. percent	Adjusted differences (CI)	Unadj. percent	Adjusted differences (CI)
DHP						
Baseline	5.78 (4.39)	− 1.90	0.30	− 0.06	0.18	− 0.04
Year 1	3.86 (4.09)	(− 2.24, − 1.57)	0.25	(− 0.10, − 0.02)	0.14	(− 0.07, 0.01)
Comparison						
Baseline	5.86 (3.75)	− 0.21	0.32	− 0.06	0.16	− 0.01
Year 1	5.65 (4.23)	(− 0.57, 0.16)	0.26	(− 0.10, − 0.02)	0.15	(− 0.04, 0.03)
Difference in differences	− 1.71	− 1.70 (− 2.19, − 1.20)	0	0 (− 0.06, 0.06)	− 0.04	− 0.03 (− 0.08, 0.01)
*p* value	–	< .001	–	.966	–	.164

The statistical model used for disease management visits employed a Poisson distribution with a log link function, and binomial logit models were used to model emergency room and hospital utilization

**Table 6:** Predicted Change with DHP Exposure, Relative to No Exposure, for the Opt-Out Samples (Difference in differences)

Utilization measure	Disease management visit count		Percent with any ER visit		Percent with any hospitalization	
Unadjusted vs. adjusted	Unadj. means (SE)	Adjusted differences (CI)	Unadj. percent (SE)	Adjusted differences (CI)	Unadj. percent (SE)	Adjusted differences (CI)
DHP						
Baseline	5.80 (4.46)	− 1.87	0.29	− 0.03	0.16	− 0.02
Year 1	3.91 (4.09)	(− 2.21, − 1.53)	0.26	(− 0.07, 0.01)	0.14	(− 0.05, 0.02)
Comparison						
Baseline	5.94 (3.84)	0.14	0.25	0.02	0.16	− 0.02
Year 1	6.09 (4.52)	(− 0.43, 0.72)	0.27	(− 0.04, 0.08)	0.15	(− 0.06, 0.03)
Difference in differences	− 2.04	− 2.02 (− 2.68, − 1.35)	− 0.05	− 0.05 (− 0.13, 0.02)	0	0 (− 0.06, 0.06)
*p* value	–	<.001	–	.165	–	.982

The statistical model used for disease management visits employed a Poisson distribution with a log link function, and binomial logit models were used to model emergency room and hospital utilization

## DISCUSSION

Using a strong quasi-experimental study design, we evaluated the relationship between the DHP exposure and healthcare utilization among low-income beneficiaries. Our findings of a negative association between DHP exposure and disease management visits in the absence of an association with emergency room and hospital utilization make an important contribution to the literature, as this is the only study to our knowledge to assess the relationship between the DHP and healthcare utilization at the individual level and the only study to our knowledge to assess this relationship among a cohort of beneficiaries with low household incomes. The robustness of the results to sensitivity analyses conducted using only DHP-exposed beneficiaries enrolled using an opt-out strategy lends credibility to the findings by lowering the likelihood that the observed results are merely a product of selection bias.

While a decline in disease management visits associated with the DHP seems somewhat counterintuitive, plausible explanations include reduced disease management visits in the setting of reduced clinical need and/or substitution of some disease management visits with telephone case management visits. However, we lack data points such as HbA1c values and the frequency of telephone case management visits that could help to further elucidate the mechanisms driving these results. An alternative explanation for these results is reduced access to care driven by the DHP. However, this explanation is less likely given that there is no accompanying increase in emergency room utilization. Furthermore, the positive association between the DHP and oral hypoglycemic medication adherence among beneficiaries with low household incomes (< $30 K annually) and low baseline medication adherence found by Narain et al. makes this explanation less likely.^17^ With respect to the emergency room and hospitalization findings, we suspect that a relatively short study time horizon may have contributed to the null results. Emergency room and hospital utilization among diabetes patients typically stems from the complications of chronic diseases associated with long-standing diabetes such as congestive heart failure and coronary artery disease.^23^ The prevalence of congestive heart failure and coronary artery disease for individuals in our study sample is only roughly 3% and 10% respectively.

Our results for ER utilization diverge from that of the Moin et al. study which find reductions in ER utilization associated with DHP exposure.^15^ These differences may stem from differences in the study designs. Specifically, differences between the study populations used in the Moin et al. study and this study include analysis of beneficiaries of all income levels rather than an emphasis on beneficiaries with low household incomes and inclusion of beneficiaries with both pre-diabetes and diabetes while we restrict our sample to beneficiaries with diabetes. Additionally, the Moin et al. study findings are based on an employer-level analysis rather than an individual-level one. As such, they propensity score match at the employer level while we propensity score match at both the employer and individual levels.

The study results must be viewed in the context of some important limitations. A key underlying assumption of the DID approach is that secular time trends for the DHP-exposed and comparison beneficiaries do not differ in the pre-treatment time period. We use propensity score matching in an effort to ensure similarity of pre-treatment secular time trends across the DHP-exposed and comparison beneficiaries, but this strategy does not account for differences in unmeasurable factors. Our study approach also reflects an intent-to-treat design which may result in beneficiaries without DHP insurance coverage being included in the treatment population. Consequently, DHP effect estimates may be biased towards the null. However, this intent-to-treat design reduces the risk of selection bias being a plausible explanation for our results. Additionally, given that our data source is claims data, we lack data on health outcomes that could be valuable for providing context to the study results. Lastly, our results also reflect average effect estimates for the DHP. As such, effects may vary across DHP implementation strategies.

We do not find changes in emergency room and hospital utilization among DHP-exposed beneficiaries with low household incomes. These null findings may be attributable to a relatively healthy and young study population in addition to a relatively short duration of follow-up.^9^ In light of the DHP-associated benefits for medication adherence found by Narain et al., studies with a larger sample size, which can stratify populations across co-morbidity level and which can follow DHP-exposed and comparison beneficiaries over a longer time horizon, may find more favorable results.^17^ Nonetheless, we do find a negative association between DHP exposure and reduced disease management visits which may engender some cost savings that can be used to offset the cost of implementing the program. Additionally, fewer in-person disease management visits may translate into less missed work and higher levels of employee productivity, potentially leading to indirect cost savings for employers associated with the DHP in a relatively short time frame.^24^

## CONCLUSION

We used strong quasi-experimental studies and administrative/pharmacy claims data to evaluate the effect of the Diabetes Health Plan (the first disease-specific health plan based on value-based health insurance benefit principles) on healthcare utilization among beneficiaries with household income ≤ $30,000 and found a negative association between DHP exposure and disease management visits but no relationship with emergency room or hospital utilization. Future studies are needed to determine if the associated reduction in disease management visits reflects improved diabetes management or reduced access to care.
